# Effect of cyclophospamide, doxorubicin, vincristine and prednisone/prednisolone (CHOP) chemotherapy regimens on left ventricular systolic function and cardiac structural abnormalities in non-Hodgkin lymphoma patients

**DOI:** 10.1186/s43046-025-00288-w

**Published:** 2025-05-01

**Authors:** Imanita Septianda, Mochamad Yusuf Alsagaff, Budi Susetyo Pikir, Ami Ashariati, Muhamad Robiul Fuadi, Fatimah Zahra

**Affiliations:** 1 Faculty of Medicine, Airlangga University, Surabaya, Indonesia; 2 Dr. Soetomo General Academic Hospital, Surabaya, Indonesia

**Keywords:** Cancer, Cardiotoxicity, Chemotherapy, Non-Hodgkin lymphoma

## Abstract

**Background:**

The cyclophospamide, doxorubicin, vincristine, and prednisone/prednisolone (CHOP) regimen, a standard treatment for aggressive non-Hodgkin lymphoma (NHL), is effective but linked to chemotherapy-induced cardiotoxicity (CDIC), such as heart failure and left ventricular dysfunction. Anthracyclines like doxorubicin are key contributors to CDIC. This study examines left ventricular dysfunction and structural abnormalities in NHL patients receiving CHOP therapy with cumulative doxorubicin doses ≥ 250 mg/m^2^, aiming to improve early CDIC detection and guide timely cardioprotective interventions.

**Methods:**

This prospective cohort study was conducted at Dr. Soetomo Regional General Hospital, Surabaya, from June to October 2023. We included NHL patients aged 18–60 years at any stage, treated with CHOP regimens containing cumulative doxorubicin doses ≥ 250 mg/m^2^, who completed at least four chemotherapy cycles, had baseline left ventricular ejection fraction (LVEF) ≥ 50%, and good echocardiographic window quality. Data were collected from medical records, biochemical tests, and echocardiography. Wilcoxon and paired *T*-tests were used for statistical analyses. The primary outcome was cardiac function, evaluated by reductions in LVEF, increased left ventricular strain, new structural abnormalities, and elevated high-sensitivity (HS) troponin and NT-proBNP levels post-chemotherapy.

**Results:**

A total of 32 patients, aged 46.62 ± 16.64 years, were included, with the colli region and stage 2 NHL being the most common. Significant differences were observed in all parameters between pre- and post-chemotherapy. LVEF decreased by − 2.40% ± 1.79 (*p* < 0.001), while global longitudinal strain (GLS) declined by 2.25 ± 1.36 (*p* < 0.001). HS troponin I levels showed a significant increase of 17.27 ± 36.29 (*p* < 0.001), and NT-proBNP levels rose by 321.23 ± 85.34 (*p* < 0.001). The study also identified a higher risk of cardiotoxicity in patients over 50 years of age.

**Conclusion:**

The CHOP regimen poses significant risks of subclinical cardiac dysfunction and structural abnormalities in NHL patients, with cumulative doxorubicin dosage being a key contributor. Early detection using sensitive biomarkers like GLS and NT-proBNP is crucial for identifying cardiotoxicity and enabling timely interventions.

**Supplementary Information:**

The online version contains supplementary material available at 10.1186/s43046-025-00288-w.

## Background

Non-Hodgkin lymphomas (NHL) are a diverse group of lymphoproliferative malignancies characterized by greater unpredictability compared to Hodgkin lymphomas and a significantly higher tendency to spread to extranodal sites [1]. NHL ranks as the 11 th most frequently diagnosed cancer and the 11 th leading cause of cancer-related deaths worldwide. In 2020, an estimated 544,352 individuals were diagnosed with NHL, resulting in 259,793 deaths globally [2]. In Indonesia, according to the Indonesian National Coordinating Board for Hematology Medical Oncology Internal Medicine, NHL ranks as the sixth most common cancer and the third fastest-growing malignancy, claiming hundreds of thousands of lives [3].

For cancer patients, conventional chemotherapy remains the preferred course of treatment [4]. When it comes to first-line chemotherapy therapy for aggressive NHL patients, the cyclophosphamide, doxorubicin, vincristin, prednisone/prednisolone (CHOP) regimen is considered the gold standard due to its high therapeutic success rates and enhanced survival rates [4]. Regretfully, in practical application, chemotherapy-induced cardiotoxicity (CDIC) continues to be a significant issue [4]. According to recent studies, cardiovascular events that happen sooner in this population are the reason for an increase in morbidity [4]. The most common CDIC events are heart failure and/or left ventricular dysfunction. The two primary anticancer agents frequently associated with left ventricular dysfunction are anthracyclines and targeted therapies [4].

The European Society of Cardiology recommends various diagnostic procedures for individuals who have received anthracycline doses of ≥ 250 mg/m^2^, including electrocardiography, echocardiography, and blood tests for troponin and natriuretic peptides [5]. A preliminary study by Prayogo et al. reported significant differences in ejection fraction and high-sensitivity troponin T (hsTnT) levels between the fourth pre-chemotherapy and post-chemotherapy cycles [6]. In NHL patients undergoing CHOP therapy, hsTnT and ejection fraction can be used synergistically to enhance the detection of cardiotoxicity, providing complementary diagnostic coverage [5, 6].

A study by Avila et al. demonstrated that combining a low global longitudinal strain (GLS) value (< 17%) with a brain natriuretic peptide (BNP) serum concentration > 17 pg/mL significantly enhanced the accuracy of early cardiovascular event detection months after chemotherapy [7]. To identify subclinical anthracycline-induced cardiotoxicity at the earliest possible stage—approximately 6 weeks after the initial dose—in asymptomatic female breast cancer patients, Sulaiman et al. utilized a relative reduction in GLS paired with a relative increase in N-terminal pro B-type natriuretic peptide (NT-proBNP) [8]. Several studies advocate for simultaneous evaluation of GLS and serum troponin levels [9, 10]. However, echocardiographic examination has limitations, including being operator-dependent, requiring extended examination times, and often involving long waiting periods, as observed at Dr. Soetomo Hospital in Surabaya.

This research aims to determine the average degree of left ventricular dysfunction and new structural abnormalities detected through echocardiography when combined with laboratory tests, following chemotherapy with a CHOP regimen containing doxorubicin at total doses of ≥ 250 mg/m^2^ in individuals with NHL. Furthermore, this study seeks to identify the early onset of CDIC to assist clinicians in determining the optimal timing to initiate cardioprotective preventive therapy during the subclinical phase of cancer therapy-related cardiac dysfunction (CTRCD). This approach is expected to improve patient outcomes and reduce overall treatment costs.

## Methods

### Design and population

This prospective cohort study employs observational analysis and was conducted at the outpatient unit of Dr. Soetomo Regional General Hospital, Surabaya, between June and October 2023. The study group consisted of NHL patients at various stages who were referred to the cardiology outpatient unit and provided informed consent for participation. The inclusion criteria were as follows: (1) NHL patients aged 18–60 years, at any stage, treated with CHOP regimens containing a cumulative doxorubicin dose of ≥ 250 mg/m^2^, (2) completion of at least four cycles of chemotherapy, (3) no history of prior chemotherapy, (4) baseline EF of ≥ 50%, (5) moderate cardiotoxicity stratification, and (6) good echocardiographic window quality. While the exclusion criteria include the following: (1) NHL involving the mediastinum, (2) history of moderate to severe valvular heart disease, congenital heart disease, or coronary artery disease, (3) history of diabetes mellitus or chronic kidney disease, and (4) body mass index (BMI) > 30 kg/m^2^.

All participants underwent echocardiography and cardiac biomarker testing 1 week before and 3 months after chemotherapy. The study’s primary outcome was cardiac function, assessed by left ventricular ejection fraction (LVEF) reductions, increased left ventricle strain, new structural abnormalities identified via echocardiography, and biomarker levels such as HS Troponin and NT-pro BNP following the chemotherapy regimen. Additional data, including demographic variables such as age, BMI, blood pressure, comorbidities, chemotherapy regimen, and dosage, were collected retrospectively from medical records. Confounding variables in this study included the doses of other chemotherapy agents, specifically cyclophosphamide, vincristine, and prednisone/prednisolone.

### Statistical analysis

The research data were processed using SPSS version 26.0. Univariate analysis was performed to explore the characteristics of each variable, with results presented descriptively in frequency distribution tables. Bivariate analysis was conducted using the Wilcoxon Sign Rank test for non-normally distributed data and the paired *T*-test for normally distributed data. The McNemar test was used to analyze newly identified structural heart abnormalities. A *p*-value of less than 0.05 was considered statistically significant.

### Ethical statement

This study was approved by the Research Ethics Committee of Dr. Soetomo General Hospital, with author MYA as the principal investigator (protocol number: 0685/KEPK/VI/2023). It was conducted in compliance with international ethical standards for research involving human participants. All participants provided written informed consent, ensuring confidentiality and the exclusion of personally identifiable information.

## Results

### Baseline characteristic

A total of 32 patients met the inclusion criteria for this study. Of the study population, 53.6% were female. Patients were assessed after completing an average of five chemotherapy cycles, with a cumulative doxorubicin dose of 275 mg/m^2^. The mean age of the participants was 46.62 ± 16.64 years, ranging from 21 to 77 years. The age group with the highest representation was 20–30 years, comprising 8 participants (25%), while the > 70 age group had the fewest participants, with 3 individuals (9.38%).

Samples from 11 different NHL regions were collected from the study population, including the tonsil, nasopharynx, ocular, inguinal, submandibular, ileum, caecum, axilla, and hypopharynx. The colli region contributed the largest number of samples, with up to 10 patients (31.3%). Stage 2 non-Hodgkin lymphoma had the highest representation, with 13 samples (40.63%), followed by stage 3 with 11 samples (34.47%), stage 4 with 5 samples (15.63%), and stage 1 with 3 samples (9.38%).

Concomitant hypertension was observed in 9.4% of patients, with three individuals having a history of treatment with the antihypertensive drug amlodipine. None of the samples had a history of stroke. The highest recorded BMI in the sample was 27.68 kg/m^2^, with an average BMI of 21.30 ± 2.19 kg/m^2^. Among the samples, 3 were underweight, 1 had grade I obesity, and 28 had a normal body mass index, as detailed in Table 1.

**Table 1:** Basic characteristics of the subjects of the study

Variables	*N* (%)	Mean ± SD
Age	NA	46.62 ± 16.64
Male patients	15 (46.32%)	NA
Hypertension	3 (9.34%)	NA
BMI	NA	21.30 ± 2.19
Sinus rhythm	32 (100%)	NA
Baseline heart rate	NA	89.63 ± 13.4
Accumulative doxorubicin dosage	NA	275
Total chemotherapy cycles	NA	5

*BMI* body mass index.

Table 2 summarizes the echocardiography findings before and after chemotherapy, showing an increasing trend in several abnormalities. These include trivial mitral regurgitation, trivial and mild tricuspid regurgitation, trivial pulmonary regurgitation, LV concentric remodelling, and minimal pericardial effusion following chemotherapy. Notably, no stenosis abnormalities were detected in the mitral, aortic, tricuspid, or pulmonary valves either before or after chemotherapy.

**Table 2:** The abnormalities found in heart valves and chamber dimensions before and after chemotherapy

Abnormality	Pre-chemotherapy	Post-chemotherapy
Mitral valve		
Mitral regurgitation (trivial)	3 samples (9.4%)	4 samples (12.5%)
Mitral regurgitation (mild)	2 samples (6.3%)	2 samples (6.3%)
Tricuspid valve		
Tricuspid regurgitation (trivial)	3 samples (9.4%)	5 samples (15.6%)
Tricuspid regurgitation (mild)	0 samples (0%)	1 sample (3.1%)
Aortic valve		
Aortic regurgitation (mild)	1 sample (3.1%)	0 samples (0%)
Pulmonary valve		
Pulmonary regurgitation (trivial)	0 samples (0%)	3 samples (9.4%)
Heart chamber dimensions		
LV concentric remodelling	3 samples (9.4%)	4 samples (12.5%)
Concentric LVH	2 samples (6.3%)	2 samples (6.3%)
Pericardial effusion (minimal)	5 samples (15.6%)	6 samples (18.8%)
Pericardial effusion (moderate)	1 sample (3.1%)	0 samples (0%)

*LV* left ventricle, *LVH* left ventricular hypertrophy.

From a total of 32 NHL patients included in the study, physical examinations, cardiac biomarker tests, and echocardiography were performed, followed by risk stratification using HFA-ICOS scores. All patients were classified into the medium-risk group, with scores ranging from 2 to 4. The highest HFA-ICOS score of 4 was observed in 2 patients (6.25%), a score of 3 was found in 5 patients (15.63%), and the lowest score of 2 was present in 25 patients (78.13%).

### Normality test and clinical parameter analysis

After performing data normality testing using the Shapiro–Wilk test, it was found that the LVEF, HS troponin I (hsTnI), and NT Pro BNP data were not normally distributed (Shapiro–Wilk *p*-value > 0.05). Therefore, these data were analyzed using the Wilcoxon Signed-Rank test. For the normally distributed GLS data (Shapiro–Wilk *p*-value < 0.05), a paired *T*-test was applied. The result of the normality test is shown in Supplementary Table 1. Table 3 shows that, upon conducting the Wilcoxon Signed-Rank test for EF, hsTnI, and NT-proBNP, the results revealed significant differences (*p* < 0.05) between the pre- and post-cumulative dose of doxorubicin ≥ 250 mg/m^2^. The paired *t*-test on the GLS parameter also showed a statistically significant difference (*p* < 0.001) in the group of NHL patients who received a 5-cycle CHOP regimen with a cumulative doxorubicin dose of ≥ 250 mg/m^2^.

**Table 3:** Analysis of research variables pre- and post-accumulative dose of chemotherapy

Variables	*N*	Pre			Post		Mean difference	*P* value
		**Mean**	**SD**		**Mean**	**SD**		
LVEF	32	66.19%		3.58	63.78%	3.31	− 2.40% ± 1.79	< 0.001**
GLS (median)	32	− 18.78		2.62	− 16.53	2.76	2.25 ± 1.36	< 0.001*
hsTnI	32	8.78		22.68	26.06	42.88	17.27 ± 36.29	< 0.001**
NT-ProBNP	32	117.30		292.19	438.55	89.23	321.23 ± 85.34	< 0.001**

*GLS* global longitudinal strain, *hsTnI* high-sensitivity troponin I, *LVEF* left ventricular ejection fraction, *NT-proBNP* N-terminal pro B-type natriuretic peptide, *SD* standard deviation.

Before chemotherapy, the highest LVEF value recorded was 79%, while the lowest was 61%. After chemotherapy, the highest LVEF value decreased to 75%, and the lowest to 58%. Among the samples, 3 patients (9.4%) did not experience any decrease in LVEF. On average, the percentage reduction in LVEF was 3.5% ± 3.58 from the baseline, with the greatest reduction reaching 8.6%. The mean LVEF post-chemotherapy was 63.78% ± 3.31, which remains within the normal range (50–70%), despite a statistically significant reduction.

Regarding GLS, pre-chemotherapy values ranged from − 23 to − 13, while post-chemotherapy values ranged from − 22 to − 11. Only 3 patients (9.4%) did not show a reduction in GLS following chemotherapy. The average percentage decrease in GLS value was 9.8% ± 7.40 from the baseline, with the highest reduction being 14.8% and the lowest being 0%. The mean GLS shifted to − 16.53% ± 2.76 post-chemotherapy, approaching the lower limit of the normal range (− 18 to − 24%). This suggests early signs of myocardial dysfunction, as more negative values indicate better systolic performance.

The highest pre-chemotherapy hsTnI value was 79 pg/mL, while the lowest was 1.3 pg/mL. Following chemotherapy, the highest hsTnI value was 133.9 pg/mL, with the lowest remaining at 1.3 pg/mL. Notably, 1.3 pg/mL is the minimum detectable value for hsTnI using the device employed in the Clinical Pathology Laboratory of Dr. Soetomo Hospital. A total of 9 samples (28.13%) exhibited an increase in hsTnI values beyond the normal range. For hsTnI, the significant increase post-chemotherapy approached the upper reference limit (24–30 pg/mL), indicating potential subclinical cardiac injury. However, no associated complaints, clinical symptoms, or abnormalities in physical examinations were observed.

Pre-chemotherapy NT-proBNP values ranged from 11 to 566 pg/mL, while post-chemotherapy evaluations showed values ranging from 27.9 to 4311.6 pg/mL. A total of 13 patients (40.63%) exhibited NT-proBNP levels exceeding the normal range post-chemotherapy. The mean NT-proBNP level post-chemotherapy was 438.55 ± 89.23 pg/mL, well above the normal threshold (< 125 pg/mL for individuals under 75 years), suggesting a higher risk of cardiac stress or dysfunction.

### Correlation analysis between clinical parameter and study demography

Table 4 presents the demographic correlations between age and gender with the parameters of decreased LVEF and GLS values, as well as increased biomarkers NT-proBNP and hsTnI. The correlations were analyzed using Pearson or Spearman correlation tests, depending on the data distribution. There was no significant correlation between age or gender and the LVEF and NT-proBNP parameters. However, an increase in hsTnI levels was associated with increasing age, showing a moderate correlation (correlation coefficient = 0.356, *p* = 0.045). Sub-analysis revealed a significant difference in the median increase of hsTnI between patients under 50 years and those over 50 years (*p* < 0.05), as illustrated in Supplementary Fig. 1. Additionally, a decrease in GLS values was found to be influenced by gender, with a moderate correlation (correlation coefficient = − 0.394, *p* = 0.026), where the decline in GLS was more pronounced in women.

**Table 4:** Correlation of age and gender with changes in LVEF, GLS, NT pro-BNP, and HS troponin I levels

	**Delta LVEF**		**Delta NT-proBNP**		**Delta GLS**		**Delta hsTnI**	
Confounding factors	***R***	***p***	***R***	***p***	***r***	***p***	***r***	***p***
Age	0.164	0.369	0.313	0.081	0.155	0.396	0.356	0.045
Gender	0.122	0.505	− 0.288	0.110	− 0.394	0.026	0.075	0.683

*GLS* global longitudinal strain, *hsTnI* high-sensitivity troponin I, *LVEF* left ventricular ejection fraction, *NT-proBNP* N-terminal pro B-type natriuretic peptide.

### Correlation analysis test between variables

Delta NT-proBNP was found to correlate with both delta LVEF (correlation coefficient = − 0.381, *p* = 0.031) and delta GLS (correlation coefficient = 0.385, *p* = 0.030), indicating a statistically significant relationship (*p* < 0.05) between changes in NT-proBNP and decreases in GLS and LVEF values in patients receiving a cumulative doxorubicin dose ≥ 250 mg/m^2^ as part of the CHOP regimen. In contrast, delta hsTnI showed no significant correlation with delta LVEF or delta GLS (*p* > 0.05), as detailed in Supplementary Table 2.

## Discussion

Recent study highlight that the cardiotoxic effects of anthracyclines, including doxorubicin, can manifest as direct vascular injury, leading to arteriolar thickening and reduced blood flow [11]. These effects are cumulative and dose-dependent [11]. Additionally, several risk factors are associated with an increased likelihood of anthracycline-induced cardiotoxicity, including intravenous bolus administration, female gender, hypertension, diabetes mellitus, and pre-existing cardiovascular disease [11].

### Structural abnormalities and cardiac dimensions

In our study, post-chemotherapy echocardiographic evaluations revealed new structural abnormalities in 18.75% of patients, with 3.13% showing worsening valve abnormalities. Additionally, abnormalities in heart chamber dimensions were observed in only 1 sample (3.13%) after chemotherapy. These findings align with Zahler et al., who reported that Anthracycline therapy is associated with new valve abnormalities, particularly mitral regurgitation [12]. This highlights the importance of regularly monitoring valve function in cancer patients [12]. While chemotherapy agents do not directly affect heart valves, valve disease in cancer patients can result from pre-existing lesions, radiation therapy, severe infections, or CTRCD [13]. Advanced malignancies may also lead to non-bacterial thrombotic endocarditis (marantic endocarditis), causing valve abnormalities [13]. Secondary valve regurgitations, such as mitral or tricuspid regurgitation, often occur in advanced stages of CTRCD due to ventricular dysfunction and remodeling [13].

### Left ventricular ejection fraction (LVEF)

CTRCD is typically defined as a ≥ 10% reduction in LVEF from baseline or an LVEF < 50% [14]. In our study, all patients showed a decline in LVEF, but none exceeded the 10% threshold, with the largest percentage difference being 8.6%. These findings are consistent with Prayogo et al., who reported significant LVEF reductions without clinical heart failure symptoms [6]. Subclinical LVEF reductions may reflect early myocardial dysfunction, as a decrease > 4% after a cumulative doxorubicin dose of 200 mg/m^2^ has been shown to predict cardiotoxicity with high sensitivity and specificity [15].

Our sub-analysis revealed a significant correlation between decreases in GLS and LVEF. While LVEF can be calculated without geometric assumptions using 3D datasets, its accuracy depends heavily on image quality, making it less reliable for detecting minor regional myocardial changes and prone to variability [16]. Chemotherapy-induced myocardial dysfunction often affects specific myocardial segments, with healthy segments compensating for damaged ones, potentially masking early dysfunction and resulting in a seemingly normal LVEF [17]. Even when LVEF remains within normal limits, anthracycline administration can cause LV diastolic disturbances linked to abnormal wall motion [17]. Even when LVEF remains within normal limits, LV diastolic disturbances linked to abnormal wall motion may occur following anthracycline administration [17]. A > 10% decline in LVEF, often preceded by increased isovolumic relaxation time, may indicate advanced dysfunction, by which point effective therapy may already be delayed [13]. Therefore, early detection of left ventricular dysfunction is crucial, and GLS serves as a more sensitive parameter for identifying early myocardial changes [13].

This is supported by the SUCCOUR trial, which found that while both GLS and LVEF showed a 3% reduction in 3D-EF, cardiotoxicity was more common in the LVEF-guided group (13.7%) compared to the GLS-guided group (5.8%, *p* = 0.02) [18]. GLS offers a tighter confidence interval than LVEF, enabling earlier detection of subtle myocardial function changes [18]. This allows for earlier and potentially more effective intervention [18].

### Global longitudinal strain (GLS)

GLS has emerged as a more sensitive parameter for detecting early myocardial dysfunction. In our study, GLS values showed a significant decline post-chemotherapy, consistent with previous research [19, 20]. Similarly, Araujo-Gutierrez et al. found that an initial GLS decrease of ≥ − 18% predicted chemotherapy-related cardiac dysfunction (adjusted HR 1.17, 95% CI 1.00–1.36; *p* = 0.044 for GLS, HR 3.54, 95% CI 1.34–9.35; *p* = 0.011 for GLS decrease), with factors such as tobacco use, pre-chemotherapy systolic blood pressure, and cumulative anthracycline dose also playing a role [20].

GLS reduction > 15% is recommended by the ESC as a threshold for subclinical CTRCD diagnosis [5]. A decrease in GLS is also linked to increased cardiovascular mortality. In a study by Ali et al., involving 450 patients with hematologic malignancies, an initial GLS of less than − 17.5 was associated with a higher risk of cardiac death or symptomatic heart failure [21]. In this study, the largest decrease in GLS was 14.8%. Based on GLS parameters, no cases of CTRCD were identified in patients who received a cumulative doxorubicin dose of ≥ 250 mg/m^2^.

Our study also found a significant correlation between decreased GLS scores and gender. These findings align with research by Hung et al., which observed sex-specific cardiac remodeling patterns in elderly populations without heart failure symptoms [22]. Women demonstrated a more pronounced increase in torsion, lower E′ myocardial relaxation speed, stronger concentric remodeling, smaller LV cavity size, and greater GLS, global circumferential strain (GCS), and torsion compared to men (all *p* < 0.05) [22]. While higher torsion was generally associated with hypertension and aging, the relationship was more pronounced in women than in men [22].

Our analysis highlights two key aspects from previous studies. First, this study focused exclusively on individuals with hematologic malignancies, who were exposed to higher doses of anthracyclines from the start of treatment. Second, unlike other studies, patients with an LVEF below 55% were excluded, emphasizing the value of early GLS decline as a predictive clinical tool for detecting anthracycline-induced CTRCD in patients with normal LVEF.

### High-sensitivity troponin (hsTnI)

This study demonstrates a significant increase in hsTnI levels before and after treatment with the CHOP regimen containing doxorubicin. These findings align with Prayogo et al., who reported that 10 of 35 subjects had hsTnT levels exceeding the 99 th percentile (> 14 pg/mL), indicating myocardial injury [6]. Elevated hsTnI and LVEF collectively reflect the spectrum of cardiotoxicity, encompassing both cellular damage and functional impairment [6]. Based on hsTnI parameters, we identified mild, asymptomatic CTRCD in 9 patients (28.1%) who received a cumulative Doxorubicin dose of ≥ 250 mg/m^2^.

Cardinale et al. found that elevated Troponin I levels predict cardiovascular events, including heart failure, arrhythmias, and severe LVEF decline (< 25%) [23]. Persistent troponin I elevation (≥ 0.08 ng/mL) was associated with higher rates of heart disease (37% for transient elevations vs. 84% for persistent elevations; *p* < 0.001), and patients with positive troponin levels had a 62% risk of significant LVEF decline compared to 5% in those with negative troponin [23].Increased hsTnI levels detected ≥ 14 days post-chemotherapy suggest chronic cardiomyocyte injury, distinguishing it from acute damage [24]. Troponin I typically peaks within 24 h in 55% of patients and within 12 h in 33% of patients post-chemotherapy [24].

In our study, hsTnI elevation was associated with age, consistent with Kilickap et al., who reported increased hsTnT levels in older patients (> 44 years) over a 6-month follow-up [25]. Similarly, Díaz-Antón et al. observed a linear relationship between hsTnT and age, though toxicity levels were not directly linked to age [26].

### N-terminal prohormone of B-type natriuretic peptide (NT-pro BNP)

Anthracyclines can lead to both acute and chronic heart failure. NT-pro BNP, with its long and stable half-life and ability to accumulate at higher concentrations, can be used to detect moderate systolic or diastolic heart failure and left ventricular dysfunction even in asymptomatic patients [26]. This study found a significant increase in NT-pro BNP levels after five cycles of chemotherapy. From the NT-pro BNP parameters, 11 samples that received a cumulative doxorubicin dose of ≥ 250 mg/m^2^ showed mild, asymptomatic CTRCD. Sandri et al. reported that persistent increases in NT-pro BNP after anthracycline administration predict left ventricular dysfunction [27]. Similarly, Pichon et al. found that in 79 women receiving anthracyclines for breast cancer, BNP levels > 51.3 ng/L predicted cardiotoxicity with 83% sensitivity and 90% specificity, where cardiotoxicity was defined as an LVEF decrease observed via ventriculography [28].

Ehrhardt et al. found that among long-term childhood cancer survivors exposed to anthracycline doses > 100 mg/m^2^, abnormal GLS and elevated NT-pro BNP levels identified individuals at higher risk for future cardiomyopathy, even with an EF ≥ 50% on echocardiographic evaluation [29]. A targeted surveillance approach using GLS and NT-pro BNP is particularly beneficial for survivors at intermediate to high risk [29].

### Limitations

This prospective study investigated the cumulative dose of doxorubicin as the primary factor contributing to cardiotoxicity in non-Hodgkin lymphoma (NHL) patients receiving the CHOP regimen. Its strengths include a comprehensive assessment of cardiotoxicity using multiple biomarkers (GLS, hsTnI, NT-proBNP, and LVEF) and the identification of age as a significant risk factor, highlighting the need for personalized monitoring strategies.

However, several limitations should be acknowledged. First, the single-center design may limit the generalizability of the findings, despite the facility being the largest hospital in eastern Indonesia serving a diverse population. Multi-center studies are needed to validate and extend these results. Second, potential confounding factors, such as the effects of other CHOP regimen components (e.g., cyclophosphamide, vincristine, and prednisone/prednisolone), were not fully controlled. Third, the short-term follow-up period of 3 months, while consistent with recommendations for early cardiotoxicity monitoring, does not provide insights into whether the observed cardiac changes are transient, reversible, or indicative of long-term clinical consequences. Fourth, the use of the Alinity tool with a minimum detectable hsTnI level of 1.3 pg/mL may have limited the detection of subtle subclinical findings. Finally, delays in placing blood samples into cooling storage may have impacted biomarker analysis. Despite these limitations, our study provides valuable insights into the cardiotoxic effects of the CHOP regimen and underscores the importance of sensitive biomarkers for early detection and risk stratification.

## Conclusion

This study highlights the cardiotoxic effects of the CHOP regimen with cumulative doxorubicin doses ≥ 250 mg/m^2^ in NHL patients across various stages. While no clinical CTRCD cases were observed, significant subclinical cardiac changes were detected, including structural abnormalities, reduced GLS and ejection fraction, and elevated hsTnI and NT-proBNP levels. The study also identified an increased risk of cardiotoxicity in patients over 50, with a higher likelihood of cardiac changes within 3 months post-treatment. These findings emphasize the importance of early detection using sensitive biomarkers like GLS and NT-proBNP to identify subclinical cardiotoxicity and guide timely interventions. Further multicenter studies with larger populations and longer follow-up periods are needed to validate these findings and develop strategies to mitigate cardiotoxic risks in cancer survivors.

## Supplementary Information

Supplementary Material 1.

## Data Availability

The datasets generated during and/or analyzed during the current study are available from the corresponding author on reasonable request.

## Abbreviations

BMI: Body mass index
BNP: Brain natriuretic peptide
CDIC: Chemotherapy-induced cardiotoxicity
CHOP: Cyclophosphamide, doxorubicin, vincristine, prednisone/prednisolone
CTRCD: Cancer therapy-related cardiac dysfunction
EF: Ejection fraction
GLS: Global longitudinal strain
HFA-ICOS: Heart Failure Association-International Cardio-Oncology Society
HS Troponin: High-sensitivity troponin
hsTnI: High-sensitivity troponin I
hsTnT: High-sensitivity troponin T
kg/m^2^: Kilograms per square meter
LVEF: Left ventricular ejection fraction
LV: Left ventricular
NHL: Non-Hodgkin lymphoma
NT-proBNP: N-terminal pro B-type natriuretic peptide
pg/mL: Picograms per milliliter
SPSS: Statistical package for the social sciences
3D-EF: Three-dimensional ejection fraction
