# Supplementary material for: Effect of cyclophospamide, doxorubicin, vincristine and prednisone/prednisolone (CHOP) chemotherapy regimens on left ventricular systolic function and cardiac structural abnormalities in non-Hodgkin lymphoma patients
Source: J Egypt Natl Canc Inst. 2025 May 1;37:29. doi: 10.1186/s43046-025-00288-w (PMC13313428; doi:10.1186/s43046-025-00288-w)
Supplement: Supplementary file 1 — Supplementary Material 1. [file 43046_2025_288_MOESM1_ESM.docx]

**SUPPLEMENTARY FILES**

**Supplementary Figure 1. Scatter plot showing the correlation between delta HS Troponin I and age**

**
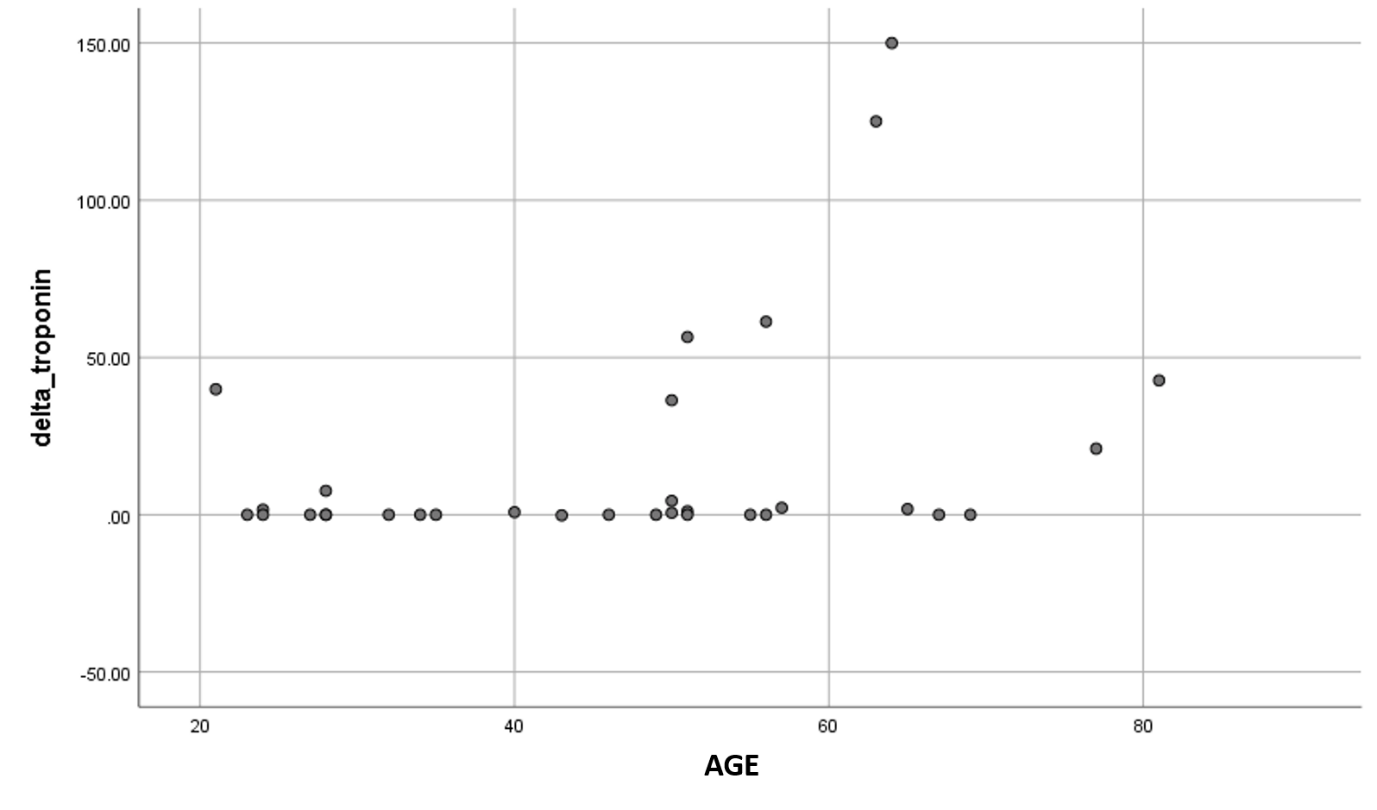
**

**Supplementary Table 1. Results of the Normality Test**

| **Variables** | **Shapiro-Wilk** | | **Data Distribution** |
| --- | --- | --- | --- |
|  | **Statistic** | **P value** |  |
| LVEF | 0.863 | 0.001 | Abnormal |
| GLS | 0.960 | 0.275 | Normal |
| HS Troponin I | 0.383 | 0.000 | Abnormal |
| NT Pro BNP | 0.330 | 0.000 | Abnormal |

**Supplementary Table 2. Results of the Correlation Test Between Variables**

| **Variables** | **Spearman Correlation** | |
| --- | --- | --- |
|  | **R** | **p** |
| Delta LVEF – Delta HS Troponin | -0.131 | 0.475 |
| Delta LVEF – Delta NT proBNP | -0.381 | 0.031 |
| Delta GLS – Delta HS Troponin | 0.312 | 0.082 |
| Delta GLS – Delta NTproBNP | 0.385 | 0.030 |
